# Improving the Flavor of Fermented Sausage by Increasing Its Bacterial Quality via Inoculation with *Lactobacillus plantarum* MSZ2 and *Staphylococcus xylosus* YCC3

**DOI:** 10.3390/foods11050736

**Published:** 2022-03-02

**Authors:** Ji Wang, Jinna Hou, Xin Zhang, Jingrong Hu, Zhihui Yu, Yingchun Zhu

**Affiliations:** College of Food Science and Engineering, Shanxi Agricultural University, Jinzhong 030801, China; 17835422765@163.com (J.W.); 18435150568@163.com (J.H.); zhangxin980523@163.com (X.Z.); 15934274740@163.com (J.H.); zhihuisxau@163.com (Z.Y.)

**Keywords:** fermented sausage, lipid oxidation, lipid hydrolysis, bacterial community, flavor compounds

## Abstract

This research aims to investigate the effects of *Staphylococcus xylosus* YCC3 (Sx YCC3) and *Lactobacillus plantarum* MSZ2 (Lp MSZ2) on lipid hydrolysis and oxidation, the bacterial community’s composition, and the volatile flavor compounds in fermented sausage. The bacterial community was examined by plate counting and high-throughput sequencing. Differential flavor compounds in non-inoculated and inoculated sausages were identified by principal component analysis (PCA) and orthogonal partial least squares discrimination analysis (OPLS-DA). The results showed that the free fatty acid (FFA) content was increased after inoculating with Sx YCC3 or Lp MSZ2. The pH, peroxide value (POV), thiobarbituric acid reactive substances (TBARS) value, lipoxygenase activity, and the counts of *Enterobacteriaceae* were lower in the inoculated sausage than in the non-inoculated sausage. The bacterial inoculation enhanced the competitiveness of *Staphylococcus* and *Lactobacillus* and restricted the growth of unwanted bacteria. The OPLS-DA revealed that (Z)-hept-2-enal, (E)-2-octenal, 1-nonanal, octanal, and 1-octen-3-ol were common differential flavor compounds that were found in the inoculated sausages but were not found in the non-inoculated sausages. A positive correlation was observed between the differential flavor compounds and the relative abundance of *Staphylococcus* or *Lactobacillus*, or the FFA content. Our results indicated that inoculation with Sx YCC3 or Lp MSZ2 can improve fermented sausages’ flavor by enhancing their bacterial quality and increasing their FFA content.

## 1. Introduction

Fermentation is a technology which is used in order to increase foods’ safety and nutritional value, prolong their shelf life, and produce unique food flavors. Fermented meat is one of the typical and popular traditional meat products in China, it has a long history and includes foodstuffs such as fermented sausage, fermented ham, and fermented fish. Traditional fermented sausage (which has undergone spontaneous fermentation without a starter culture) is processed using empirical methods and the sausage processing is accompanied by complex microbial metabolic activities [1]. Additionally, the sausage is easily contaminated by various microorganisms (including spoilage or pathogenic bacteria) that are present in the environment during processing, thus it is difficult to guarantee the flavor and safety of final products [2]. To control the quality of the fermented sausage, the addition of a starter culture has becoming increasingly necessary in making fermented sausage [3]. Lactic acid bacteria (LAB) (such as *Lactobacillus plantarum*, *Pediococcus pentosaceus*, and *Lactobacillus casei*) and coagulase-negative staphylococci (such as *Staphylococcus xylosus*, *Staphylococcus saprophyticus*, and *Staphylococcus carnosus*) tend to be used as starter cultures for the production of fermented sausage [4]. LAB can inhibit the growth of spoilage bacteria and pathogenic bacteria by reducing the pH and producing bacteriocin in the fermented sausage [5]. *Staphylococcus* plays an important role in the flavor formation of fermented meat products [6] and their nitrate reductase activity is important for color formation [7]. Li et al. [8] have reported that LAB and *Staphylococcus xylosus* can inhibit the growth of spoilage microorganisms and improve the color of the meat product. Ge et al. [9] have suggested that *Lactobacillus plantarum* NJAU-01 from Jinhua ham can improve the quality and sensory characteristics of dry fermented sausage.

The hydrolysis and oxidation of lipids play an important role in flavor formation during the fermented sausage’s ripening [10]. The formation of flavor is a very complex process containing multiple chemical reactions and enzymatic actions [11]. In general, the formation of flavor requires the following two main steps [12]. The first step is lipolysis, which can increase the concentration of free fatty acids, which are the main precursor of the flavor compound [13]. Although the endogenous lipases of meat are considered to be primarily responsible for the free fatty acids’ release, the effect of bacteria on lipolysis cannot be ignored. Microorganisms can secrete lipase during the fermentation process and participate in the lipolysis [14]. Lipase has been extracted and its activity has been detected in *Lactobacillus plantarum*, *Staphylococcus xylosus*, and *Staphylococcus warneri* bacteria that have been isolated from meat products [15]. Inoculation with *Lactobacillus sakei* L. 110 and *Staphylococcus carnosus* M72 into Spanish dry-cured sausages can significantly increase the concentration of free fatty acids [16]. The second step of flavor formation is the oxidation of free fatty acids through which volatile compounds are produced, such as acids, aldehydes, ketones, and esters [17]. The moderate oxidation of the free fatty acids contributes to the flavor development of fermented meat products. However, the excessive oxidation of the free fatty acids could cause rancidity, texture and color modification, spoilage microorganism growth, and quality deterioration in fermented sausages [18]. Therefore, inoculation with a starter culture that has lipolysis and antioxidant abilities in order to promote the release of free fatty acids and prevent the excessive oxidation of the lipids has great importance for the flavor development of fermented meat products [19,20]. The thiobarbituric acid value is reduced by inoculating with a starter culture (*Pediococcus acidilactici*, *Lactobacillus plantarum* and *Staphylococcus carnosus*) in Turkish sausage storage [21].

In our previous studies, we isolated and identified 12 bacterial strains from traditional Chinese fermented food, including 4 LAB strains, 3 *Macrococcus* strains, and 5 *Staphylococcus* strains [22]. We investigated the lipid hydrolysis and antioxidant abilities of these strains in sterilized pork pulp (the paper describing this was accepted by Scientia Agricultura Sinica, as yet it is unpublished). Among them, the *Staphylococcus xylosus YCC3* (Sx YCC3) and *Lactobacillus plantarum* MSZ2 (Lp MSZ2) had high lipolysis or antioxidation abilities. The objective of this present study is to investigate the effects of Sx YCC3 alone, Lp MSZ2 alone, and their combination (at a ratio of 1:1) on the bacterial community, lipolysis, lipid oxidation, lipid composition, and volatile compound composition that are present in fermented sausage and to assess the correlation among the bacterial abundance, lipid hydrolysis, and the volatile compounds that are present in fermented sausage with non-inoculated sausages as the control group. Our results will provide a theoretical basis for the development of new starter cultures with high levels of safety and quality.

## 2. Materials and Methods

### 2.1. Strains

Sx YCC3 and Lp MSZ2 were isolated from traditional Chinese fermented foods, identified by 16S rDNA sequencing, and stored at the Meat Laboratory Center of Shanxi Agricultural University of China. Sx YCC3 was cultured in Mannitol Salt Broth at 37 °C for 48 h and Lp MSZ2 was cultured in de Man, Rogosa and Sharpe (MRS) Broth (Qingdao Hope Bio-Technology Co. Ltd., Qingdao, China) at 37 °C for 48 h. The Mannitol Salt Broth was formulated according to the method that was reported by Chen et al. [20]. The bacterial cells were collected by centrifuging at 1000× *g* for 5 min and then the bacterial cells were washed twice with 0.85% saline water and stored at 4 °C for subsequent use.

### 2.2. Preparation of Fermented Sausage Samples

Fresh lean pork and pork back fat were purchased from JiaJiaLi market in Taigu, China and transported on ice to the Meat Laboratory Center of Shanxi Agricultural University of China (the meat was sourced from a Shanxi black pig, male, six months old). The fermented sausage was manufactured according to a previously reported method with minor modification [23]. The fermented sausage was prepared at the Meat Laboratory Center of Shanxi Agricultural University of China. The lean pork was minced through a mincer (MM-12, Shaoguan Xintongli Foodstuff Machinery Co. Ltd., Shaoguan, China) and the fat was cut into cubes of about 5 mm. The formula was as follows: lean pork (800 g), back fat (200 g), NaCl (30 g), sugar (50 g), wine (15 g), sodium erythorbate (0.5 g), and sodium nitrite (0.15 g). The samples were divided into four groups (5 kg/group), namely the CK group (without a starter culture), YCC3 group (inoculated with Sx YCC3), MSZ2 group (inoculated with Lp MSZ2), and YM group (inoculated with Lp MSZ2+Sx YCC3 at the ratio of 1:1). The final concentration of the starter culture in the 3 treatment groups was approximately 10^7^ CFU/g. After thorough mixing, the mixture was stuffed into natural pork casings (Far-Eastern Casing Company, Baoding, Hebei, China) with a diameter of approximately 3 cm. The weight and length of the final sausage was approximately 200 ± 5 g and 8 ± 1 cm, respectively. The sausages in all of the 4 groups were fermented at 32 ± 1 °C and 90% relative humidity for 2 days at a constant temperature and in a humidity incubator (LRHS-150, Yuejin medical apparatus Co. Ltd., Shanghai, China) and then ripened at 15 ± 1 °C and 70–80% relative humidity for 12 days at a constant temperature and in a humidity incubator. The water activity of all of the sample groups was lower than 0.86 when measured at day 12. During the ripening process, three fermented sausages from each group were randomly taken in order to measure the indicators on days 0, 6, and 12 and they were then stored at −80 °C for subsequent analysis.

### 2.3. Lipid Composition Analysis

#### 2.3.1. Lipid Extraction

The lipids were extracted according to the previously reported method with some modification [24]. Two grams of the minced sample was added to 20 mL chloroform-methanol (2:1 *v/v*). The samples were vortexed for 2 min on a pulsing vortex mixer, stood for 15 min, centrifuged for 10 min (5000× *g*), and filtered. The filtrate was added to 5 mL of sodium chloride solution (0.85% *w*/*v*). The mixture was centrifuged at 4 °C at 3000× *g* for 10 min. The chloroform layer was collected and dried by nitrogen gas.

#### 2.3.2. Lipid Separation

The lipid content was separated by solid phase extraction using NH_2_ aminopropyl columns [25]. The 100 mg of lipids was dissolved in 1 mL of chloroform. The solution was added onto aminopropyl columns. Then neutral lipids, phospholipids, and free fatty acids were eluted by 3 mL chloroform–isopropanol solution (2:1 *w*/*w*), 3 mL diethyl ether–acetic acid solution (1:3 *w*/*w*), and 3 mL methanol, respectively. The eluent was collected and dried with nitrogen gas. The neutral lipids, phospholipids, and free fatty acids were weighed using precision scales (Beijing Nuclover Technology Co. Ltd., Beijing, China).

### 2.4. Lipid Oxidation Analysis

The lipoxygenase (LOX) activity, peroxide value (POV), and thiobarbituric acid reactive substances (TBARS) were used in order to evaluate the lipid oxidation level of the fermented sausages.

#### 2.4.1. Lipoxygenase (LOX) Activity Determination

Two grams of the fermented sausage samples were homogenized in 15 mL of phosphate buffer (50 mM, pH 7.0) containing 1 mM dithiothreitol and 1 mM ethylenediaminetetraacetic acid, stirred for 30 min under ice cooling and then centrifuged (4 °C, 10,000× *g* for 60 min). The supernatant was filtered through fiberglass and collected as crude LOX for the enzyme activity analysis. The LOX activity of the fermented sausage was determined according to the previously reported method [26]. One unit of LOX activity was defined as the absorption increment by 1 unit per minute and the activities that were related to the samples were expressed in the form of U/g sample.

#### 2.4.2. Peroxide Value Determination

The POV of the fermented sausages was determined according to China’s official standard per the *Determination of Peroxide Value in Foods* (GB/T 5009.227-2016). The result was expressed as mmol of reactive oxygen species/kg lipid.

#### 2.4.3. Thiobarbituric Acid Reactive Substances (TBARS) Determination

The TBARS values of the fermented sausages were determined according to the previously reported method by Li et al. [27]. Five g of each fermented sausage sample was added into 15 mL trichloroacetic acid (7.5% *w*/*v*), homogenized, and filtered. The 2.5 mL filtrate was mixed with 2.5 mL thiobarbituric acid (0.02 M) in test tubes. The mixture was subjected to a boiling water bath for 40 min, then cooled to room temperature, added to 3 mL chloroform, and centrifuged at 2 °C for 10 min (2000× *g*). The supernatant was collected and its absorbance at 532 nm was determined. The TBARS value was expressed as mg/kg (or mg MDA/kg).

### 2.5. Bacterial Analysis

#### 2.5.1. Bacterial Counting

The *Staphylococcus*, LAB, and *Enterobacteriaceae* were counted on mannitol salt agar (MSA), MRS, and Violet Red Bile Dextrose Agar (VRBDA), respectively, as previously described by Lu et al. [28].

#### 2.5.2. High-Throughput Sequencing of Bacteria

The total DNA sequences of the bacteria were extracted using the CTAB method as reported by Xiao et al. [14]. The DNA’s concentration and purity were detected using POCKIT Nucleic Acid Analyzer (Shanghai Thermo Fisher Scientifc Co., Ltd., Shanghai, China). The extracted total DNA was stored at −20 °C for use. The V3-V4 region of 16S rRNA was amplified through PCR (Model: T100, Bio-Rad Laboratories Co., Ltd., Hercules, CA, USA) using primer pairs 341F (5′-CCTAYGGGRBGCASCAG-3′) and 806R (5′-GGACTACNNGGGTATCTAAT-3′). The PCR products were sequenced. The amplicon sequencing, OTUs clustering, species annotation, and alpha diversity analyses were performed as described by Zheng et al. [29].

### 2.6. Flavor Compound Determination

The flavor compound was determined using headspace solid-phase microextraction-gas chromatography-mass spectrometry (HS-SPME-GC-MS). Five grams of each fermented sausage sample was minced and put into a 20 mL headspace vial. Dichlorobenzene was added into the sample as per the internal standard. The flavor compound was determined according to the method that was reported by De Lima Alves et al. [30] with some modifications in order to obtain the compound’s Chemical Abstracts Service (CAS) accession number. This accession number was matched with the corresponding compound in the database (http://www.flavornet.org/flavornet.html (accessed on 12 October 2021)). The matched compound was identified as a certain flavor compound when the shared similarity was more than 90%. The volatile flavor compound concentration was calculated according to the following formula:

C1 = C2 × S1/S2

where C1 indicates the relative concentration of the volatile compounds; S1 represents the peak area of the volatile compounds; S2 denotes the peak area of the internal standard; and C2 represents the concentration of the internal standard.

### 2.7. Statistics Analysis

All of the measurements were performed in triplicate and the results have been expressed as the mean score ± standard deviation. The analysis of variance (ANOVA) was performed using Statistix 9.0 (Tallahassee, FL, USA) to evaluate the statistical difference (*p* < 0.05). The principal component analysis (PCA) and orthogonal partial least squares discrimination analysis (OPLS-DA) were performed in order to determine the overall differences between the groups by using SIMCA-P (Umetrics software V.13.0, Malmö, Sweden). The Pearson correlation analysis was performed in order to investigate the relationship among the bacterial abundance, FFA, and volatile compounds using R package (version 2.15.3, Robert Gentleman, The University of Auckland, New Zealand). The diagrams were plotted by OriginPro 9.0 software (OriginLab, Northampton, MA, USA).

## 3. Results and Discussion

### 3.1. Lipid Composition

Neutral lipids, phospholipids, and free fatty acids are the main components of the lipids that are found in raw meat [12]. As shown in Table 1, the content of the neutral lipids, phospholipids, and free fatty acids were 77.13 g/100 g, 18.16 g/100 g, and 4.71 g/100 g, respectively, in the raw meat. The content of the neutral lipids was decreased, but no significant difference was observed between the fermented sausage meat (in the four groups) at the end of the ripening and the raw meat (*p* > 0.05). The phospholipid content in all of the samples at the end of ripening was significantly lower than those which were found in the raw meat (*p* < 0.05). The concentration of free fatty acids at the end of ripening was significantly higher than those which were found in the raw meat (*p* < 0.05), indicating that the free fatty acids were mainly produced by the hydrolysis of phospholipids. Our results were in line with previous finding by Huang et al. [12] who reported that phospholipids were the main source of free fatty acids during the processing of bacon. In our study, the free fatty acid content in the three inoculated groups ranged from 14.13 g/100 g to 18.40 g/100 g, which was significantly higher than that which was found in the CK group (12.55 g/100 g)—especially in the case of the YCC3 group (18.40 g/100 g). Iacumin et al. [31] have found that *Staphylococcus xylosus* has high lipolytic activity, thus promoting lipid hydrolysis metabolism in order to release more free fatty acids during the processing of fermenting the sausage. Xiao et al. [14] have also found that the free fatty acid concentrations in fermented sausages that were inoculated with *Staphylococcus carnosus* and *Lactobacillus sakei* were significantly higher than those that were found in sausages that were inoculated with *Lactobacillus sakei*.

### 3.2. pH

The changes in the pH values of the fermented sausages that were measured during the ripening process are shown in Table 2. The pH of all of the fermented sausages decreased in the first 6 days of ripening, which might be due to the bacterial multiplication-induced accumulation of organic acids. After 6 days of ripening, the pH of all of the fermented sausages slightly increased, which might have been attributed to the fact that the protein was degraded by proteinases, thus producing peptides, amino acids, and amines which neutralized the organic acids [3]. On day 12, the pH value in the CK group was 5.51, which was significantly higher than that in the 3 inoculation groups (*p* < 0.05). It was noteworthy that the pH values in the MSZ2 and YM inoculation groups were lower than 5.0. The pH decrease in the inoculation groups might be attributed to the initial number of LAB that were present in the fermented sausages [32] and the accumulation of organic acids (such as lactic and acetic acids) which were derived from the carbohydrate metabolism under the action of the LAB. At a low pH value, the growth of spoilage bacteria and pathogenic microorganisms was inhibited by organic acids [9]. In addition, lipase (especially acid lipase) activity is greater at lower pH values, thus releasing more free fatty acids, eventually improving the nutritional value and flavor of the fermented sausage [10].

### 3.3. POV, TBAR Value, and LOX Activity

The POV value indicates the content of hydroperoxide and peroxides and it also reflects the degree of primary oxidation of the lipids [33]. As shown in Table 2, the POV in the raw meat was 0.83 mmol/kg and it then dramatically increased (ranging from 2.40 mmol/kg to 3.67 mmol/kg) in all of the fermented sausages by the end of ripening, indicating that hydroperoxide formation rate was faster than its decomposition rate. The CK group (the non-inoculated sausage) exhibited a significantly higher POV than the 3 inoculated groups (*p* < 0.05), indicating that Sx YCC3 and Lp MSZ2 could inhibit the accumulation of hydroperoxide and peroxides. The possible reason for this result lies in that LAB and *Staphylococcus xylosus* can produce antioxidant enzymes (such as catalase and superoxide dismutase) which decompose hydroperoxide and peroxides into water, thus they have antioxidant ability [15,34].

The TBARS value is a suitable indicator for evaluating the degree of lipid oxidation during the fermentation process and this value is mainly used to assess the formation of the secondary products of oxidation [10]. Secondary products are generated through the degradation of hydroperoxides that are derived from the oxidation of unsaturated fatty acids. As shown in Table 2, the TBARS value in the raw meat was 0.11 mg/kg but it was increased in all of the fermented sausages during the ripening process, which might be due to the enzymatic oxidation and auto-oxidation of the lipids during the ripening process [26]. The TBARS values were significantly different between the inoculated sausages and the CK sausage during the ripening process. After ripening, the TBARS value in the 3 inoculated groups ranged from 0.23 mg/kg to 0.27 mg/kg, which was significantly lower than that of the CK group (0.35 mg/kg). This result suggests that Sx YCC3 and Lp MSZ2 could inhibit lipid oxidation, which is in line with a previous report by Hu et al. [35] that lipid oxidation was repressed in fermented silver carp sausages by the process of inoculating them with *Lactobacillus plantarum* and *Staphylococcus xylosus*. Consistently, Chen et al. [10] also found that the *Pediococcus pentosaceus*, *Lactobacillus curvatus*, *Lactobacillus sakei*, and *Staphylococcus xylosus* bacteria could inhibit lipid oxidation in Harbin dry sausage. It has been reported that *L. plantarum* and *L. pentosus* can produce antioxidant peptides which replace free radicals with unsaturated fatty acids and block the chain reaction of free radicals, thereby inhibiting lipid oxidation [36].

LOX is an enzyme that promotes lipid oxidation and it is a type of protein containing non-heme iron. According to Wang et al. [33], the LOX enzymatic activation process is as follows: the peroxyl radicals are reduced by Fe^2+^ on the LOX which then generates hydroperoxides and then the Fe^2+^ on the LOX is oxidized to Fe^3+^. As shown in Table 2, the LOX activity in the raw meat was 74.00 U/g and it then decreased significantly in all of the fermented sausages during ripening process. At the end of ripening, the LOX activity in the YCC3 (15.99 U/g), MSZ2 (8.26 U/g), and YM (9.38 U/g) groups was significantly lower than that which was found in the CK (18.42 U/g) group (*p* < 0.05). The difference in the LOX activity might be due to the inoculation with a starter culture, which further resulted in the difference in the processing conditions, such as the water activity and lipid oxidation product content (hydroperoxides) [37]. Our result was in accordance with the report by Wang et al. [33] which stated that, after the inoculation of a starter culture (*Lactobacillus sakei* and *Staphylococcus xylosus*), the LOX activity in traditional Chinese smoked horse meat sausage was lower than that in the non-inoculated sample. Gata et al. [38] reported that the LOX activity was maximal at pH 5.5. Our data showed that the pH value in the CK group ranged between 5.41–5.51 during ripening process, which was suitable for LOX activity, but the pH value in the 3 inoculation groups was lower than that which was found in the CK group. As a result, the LOX activity in the inoculation groups, especially in the MSZ2 and YM groups, was lower than that of the CK group.

### 3.4. Bacterial Counting

The counts of LAB, *Staphylococcus*, and *Enterobacteriaceae* in all of the fermented sausage are shown in Table 3. The count of LAB, *Staphylococcus*, and *Enterobacteriaceae* in the raw meat was 4.61 log CFU/g, 4.21 log CFU/g, and 4.04 log CFU/g, respectively. The LAB counts in the YCC3 group (7.42 log CFU/g), MSZ2 group (8.94 log CFU/g), and YM group (8.59 log CFU/g) were higher than that of the CK group at the beginning of ripening. This difference might be attributed to the bacterial type and amount in the starter culture. The LAB counts in all of the fermented sausages were lower at the end of ripening (day 12) than they were on day 6 during ripening process, which might be related to the low pH and low activity of water at the late stage of ripening [39]. The LAB play an important role in the ripening of the fermented sausage and they inhibit the growth of spoilage bacteria and pathogenic microorganisms by reducing the pH and producing bacteriocins [5].

The *Staphylococcus* counts in the CK, YCC3, MSZ11, and YM groups were 7.42 log CFU/g, 8.04 log CFU/g, 6.79 log CFU/g, and 7.91 log CFU/g, respectively, at day 0 of ripening. The *Staphylococcus* count was significantly lower in the MSZ2 group than in the other three groups during the ripening process (*p* < 0.05). One previous study has reported that the competitiveness of *Staphylococcus* is poorer than that of LAB. The acidification by LAB has a great effect on the growth of *Staphylococcus* and it can reduce the count of *Staphylococcus* by decreasing the pH [40]. *Staphylococcus* are considered to be flavor-producing bacteria. In this study, high counts of Staphylococcus were observed in the YCC3 and YM groups at the end of ripening, which might improve the flavor of the fermented sausage.

*Enterobacteriaceae* is usually regarded as a type of spoilage bacteria and it is known to have a negative effect on fermented sausages’ safety and quality [41]. The counts of *Enterobacteriaceae* in the CK, YCC3, MSZ2, and YM groups were increased to 5.52 log CFU/g, 4.11 log CFU/g, 4.58 log CFU/g, and 4.24 log CFU/g, respectively, at the end of ripening. The inoculation groups, especially the YM and YCC3 groups, displayed lower *Enterobacteriaceae* counts than the CK group, indicating that inoculation with Sx YCC3 or Lp MSZ2 could inhibit the growth of *Enterobacteriaceae*. Based on the high counts of *Enterobacteriaceae* in the CK group (5.52 log CFU/g) compared with the YCC3 group (4.11 log CFU/g) at the end of ripening, *Staphylococcus xylosus* quickly became the dominant bacteria in the YCC3 sausage; this dominance improved its competitiveness and also increased its ability to compete for nutrients against *Enterobacteriaceae*. In addition, the antimicrobial metabolites that are produced by *Staphylococcus xylosus* could inhibit the growth of *Enterobacteriaceae* [14]. Our result was in line with a previous report by Wang et al. [33] who found that the counts of *Enterobacteriaceae* in traditional Chinese smoked horse meat sausage that was inoculated with *Lactobacillus sakei* and *Staphylococcus xylosus* were significantly lower than those which were present in a non-inoculated control sample.

### 3.5. Bacterial Community

#### 3.5.1. Bacterial Richness and Diversity

**Figure 1:**
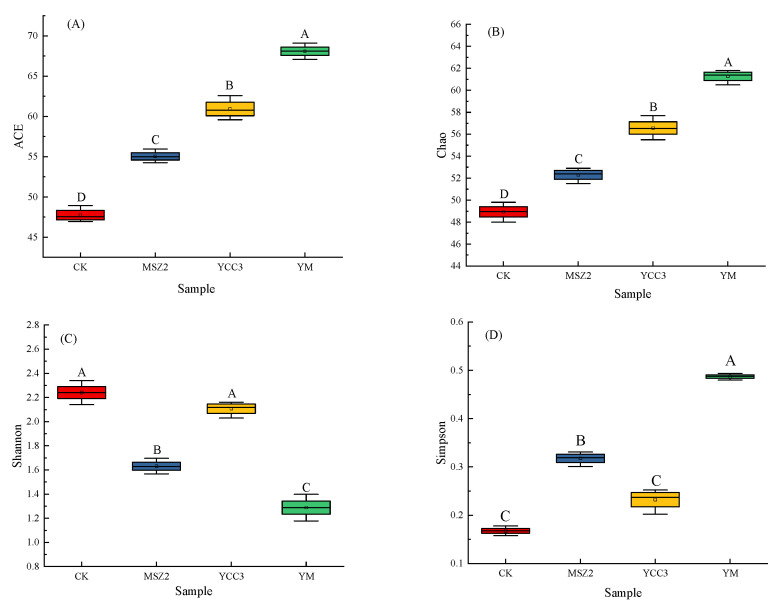
Alpha diversity in fermented sausages. Alpha diversity indexes include bacterial richness indexes (consisting of ACE and Chao 1) and bacterial diversity indexes (composed of Shannon and Simpson indexes). (**A**): ACE indexes. (**B**): Chao 1 indexes. (**C**): Shannon indexes. (**D**): Simpson indexes. CK group is red, MSZ2 group is blue, YCC3 group is yellow and YM group is green. Different letters indicate significant differences (*p* < 0.05).

The alpha diversity indexes that were used in this study include bacterial richness indexes (composed of the ACE and Chao 1 indexes) and bacterial diversity indexes (consisting of the Shannon and Simpson indexes). As shown in Figure 1, the ACE and Chao indexes were significantly higher in the three inoculation groups than in the CK group, indicating that the bacterial richness was higher in the YCC3, MSZ2, and YM groups than in the CK group. The Shannon index was higher in the CK group than it was in the inoculated groups, whereas the Simpson index was lower in the CK group than it was in the inoculated groups, suggesting that the bacterial diversity was higher in the CK group than it was in the inoculated groups due to the inhibition of the growth of unwanted bacteria. These results show that inoculation with a starter culture could reduce bacterial diversity and increase bacterial richness.

#### 3.5.2. Bacterial Composition

**Figure 2:**
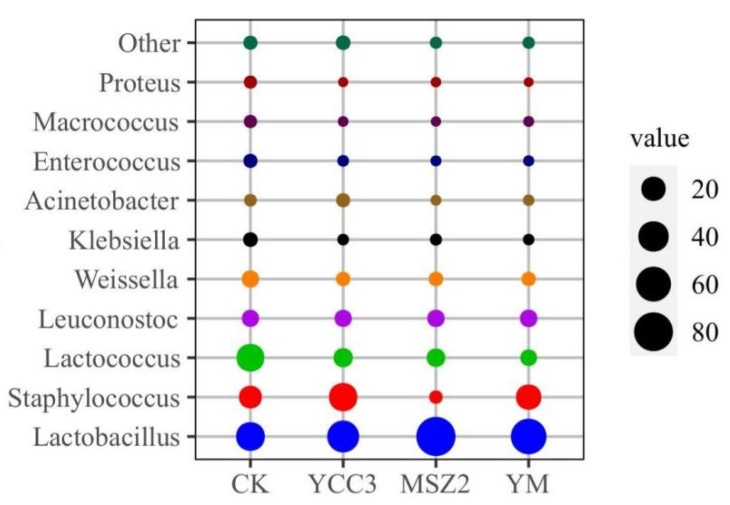
Bubble plot of bacterial relative abundance at genus level of fermented sausage. Different colors indicate different bacteria, the size of the circle indicates the bacteria’s relative abundance.

**Figure 3:**
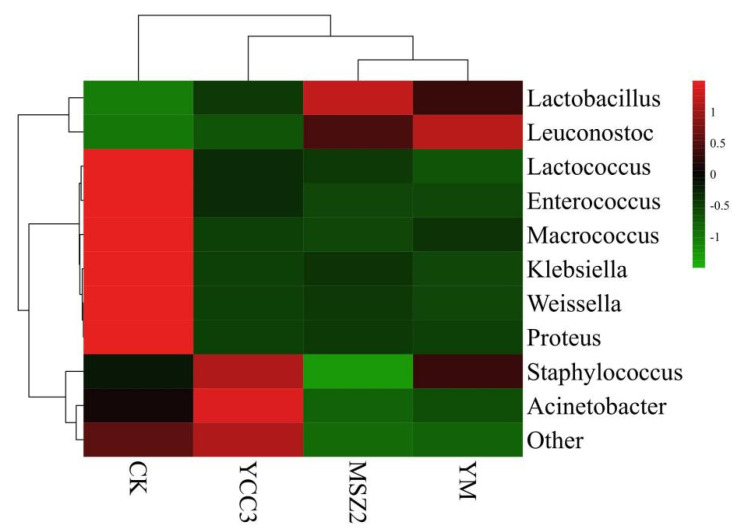
Bacterial community composition heatmap at genus level in fermented sausage. The colors correspond to normalized mean levels from low (green) to high (red).

At the end of ripening, the relative abundance of bacteria in the fermented groups at the genus level is shown in Figure 2. The bacterial community compositions in the inoculated groups were different from that of the CK group (Figure 3). *Lactobacillus*, *Lactococcus*, *Staphylococcus*, *Weissella*, *Leuconostoc*, *Klebsiella*, *Enterococcus*, and *Macrococcus* were the dominant bacteria in the CK group, whose relative abundance was 33.88%, 30.46%, 16.13%, 5.32%, 5.18%, 2.30%, 1.81%, and 1.18%, respectively. Our results were consistent with a report on traditional dry sausages from Northeast China [42].

The relative abundance of *Lactobacillus* in the inoculated groups (46.56–81.99%) was higher than that which was found in the CK group. The relative abundance of *Staphylococcus* in the YCC3 group (32.60%) and YM group (22.96%) was significantly higher than that which was found in the CK group (16.13%). However, the relative abundance of *Staphylococcus* in MSZ2 group (1.31%) was significantly lower than that which was found in the CK group (16.13%), which might be due to the inhibition of *Staphylococcus* growth that was caused by Lp MSZ2. The results of our bacterial composition analysis indicated that LAB and *Staphylococcus* predominated in the fermented groups. LAB contribute to the acidification of the sausage, resulting in a lower pH level, which ensures the safety of the sausage product. In addition, LAB also inhibit the growth of spoilage bacteria and pathogenic bacteria by producing secondary metabolites such as bacteriocin. *Staphylococcus* is considered to be a type of important bacteria for the flavor formation of fermented meat products since it can produce protease, lipase (promoting flavor generation), and antioxidant enzymes (inhibiting the rancidity of the product) [43].

*Macrococcus*, *Klebsiella*, and *Enterococcus* are usually regarded as unwanted bacteria in fermented meat products [14]. Especially, *Enterococcus* belong to a family of pathogenic bacteria which are known for threatening food safety [41]. In addition, these bacteria are considered to be a major factor in the genesis of rancidity and an off-flavor in fermented sausage [14]. These bacteria might be derived from the raw meat or the environment. In this study, the relative abundances of *Macrococcus* (0.06–0.17%), *Klebsiella* (0.32–0.53%), and *Enterococcus* (0.18–0.38%) were significantly lower in the inoculated groups than those which were found in the CK, indicating that these unwanted bacteria were inhibited by inoculating Sx YCC3 or Lp MSZ2 into the fermented sausage. Fermented sausages are easily contaminated by spoilage bacteria that are present in the environment during the production process, leading to the deterioration in the quality of the sausage [43].

In our study, the bacterial composition was changed by inoculation with Sx YCC3 and Lp MSZ2. This result was in line with that which was reported by Cardinali et al. [44], who found that the bacterial composition was different between sausages that were inoculated with a commercial starter culture (*Pediococcus pentosaceus* and *Staphylococcus xylosus*) and non-inoculated sausages. Our data have indicated that the competitiveness of LAB and *Staphylococcus* was improved by inoculating with these bacteria and that the growth of unwanted bacteria was restricted, which contributed to improving the quality and safety of the resultant fermented sausages.

### 3.6. Volatile Compounds

#### 3.6.1. Volatile Compound Composition

**Figure 4:**
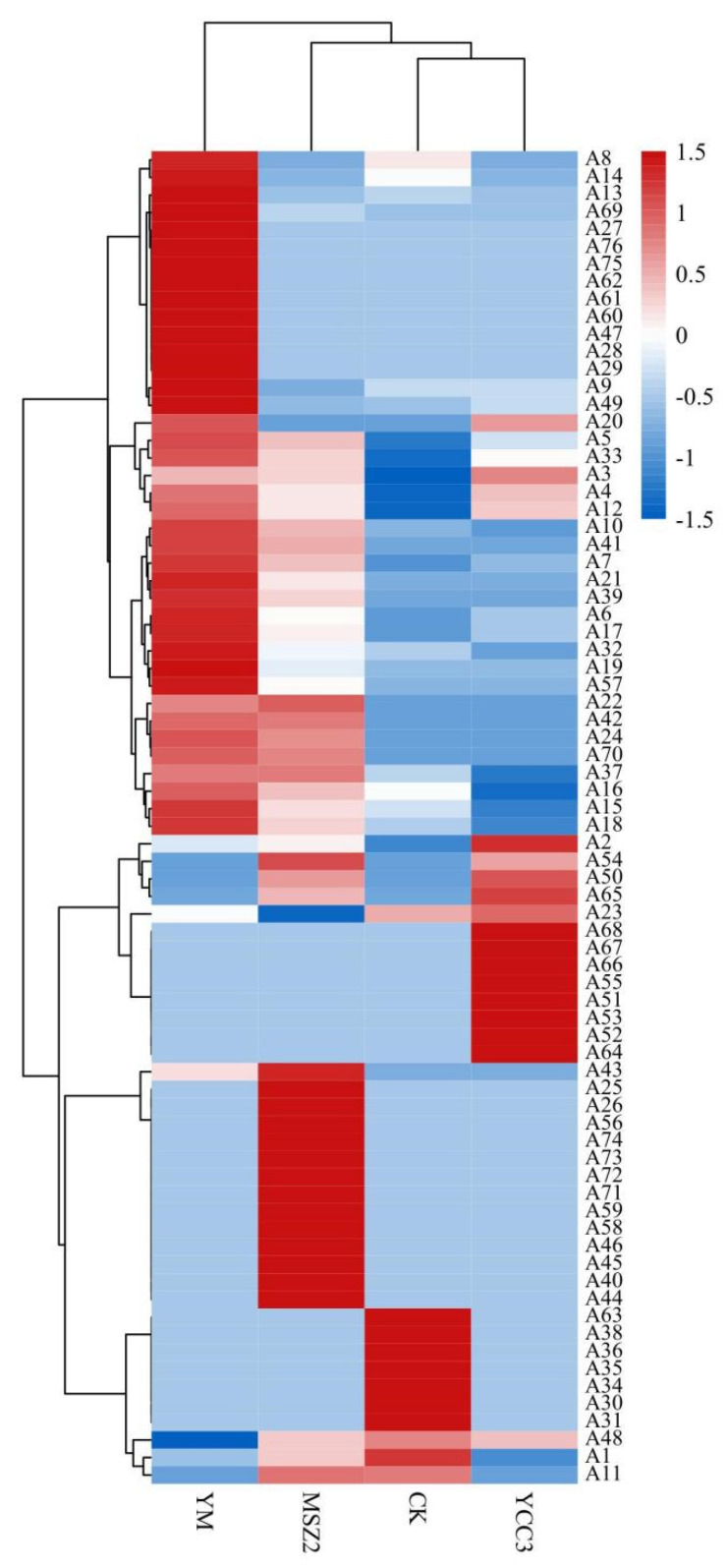
Heatmap of volatile flavor compounds in fermented sausage. The colors correspond to normalized mean levels from high (red) to low (blue).

A total of 76 volatile compounds, consisting of 22 aldehydes, 14 acids, 7 ketones, 18 alcohols, and 15 esters, were detected within the fermented sausages (Table 4). The acids, aldehydes, ketones, alcohols, and esters significantly differed in composition among the four types of fermented sausages (Figure 4), indicating that inoculation with a starter culture could change the composition of the volatile compounds that were present in the fermented sausages.

Aldehyde is one of the important flavor compounds which is found in fermented meat products and it has low flavor threshold value [45]. The content of aldehydes such as 2-undecenal, (E)-2-octenal, octanal, lily aldehyde, and (E,E)2,4-decadienal in all of the three inoculated groups was higher than that which was found in the CK group. These aldehydes are the precursors of low-molecular-weight volatile compounds and make an important contribution to the flavor formation of fermented sausages [42]. Nonanal and hexanal endow fermented meat with green grass and fat odors and these two flavor compounds are derived from the oxidation of linoleic acid and linolenic acid. They are considered to be important types of aldehydes [20]. However, hexanal is associated with lipid oxidation, thus it is used to evaluate the lipid oxidation degree, and high levels of hexanal can cause an off-flavor in food product [14]. In this study, the hexanal content was significantly higher in the CK group (304.81 μg/kg) than it was in the inoculated groups (181.89–253.75 μg/kg), which coincided with the TBARS value. Our results were consistent with the findings of Chen et al. [10] who reported that inoculating bacteria into Harbin dry sausage inhibited lipid oxidation, thereby improving the sausages’ flavor.

Ketone has low flavor threshold value and it can impart fruity, rose, floral, and tea odors to fermented sausage [45]. Ketone is formed due to the incomplete β-oxidation of free fatty acids and amino acid catabolism. The formation of ketones is closely related to the presence of *Staphylococcus* and LAB [10,42]. In this study, a total of seven ketones were detected from within all of the four fermented sausage groups. However, only one ketone (1,6-dioxacyclododecane-7,12-dione) was detected within the CK group and its concentration was significantly lower than those which was present in the inoculated sausage groups (*p* < 0.05). In addition, 1-octen-3-one, 2-undecanone, and 2-tridecanone were only detected in the YM group.

Alcohol is formed from the degradation and oxidation of lipids [46]. Compared with aldehyde, alcohol has a higher flavor threshold value and less influence on sausage flavor. However, as precursors of aldehyde and ketone, alcohol has an important effect on flavor formation [11]. In this study, a total of 18 alcohols were detected from within all of the fermented sausages, of which 1-hexanol, 1-octen-3-ol, and n-heptanol were the key flavor compounds since they had relatively low threshold values. The inclusion of 1-octen-3-ol can afford mushroom-like odor and it is indirectly produced from the oxidation of linoleic acid or other polyunsaturated fatty acid [47]. Our data showed that the content of 1-hexanol, 1-octen-3-ol, 2-pentadecyn-1-ol, and phenylethyl alcohol was lower in the CK group than it was in the inoculated groups, indicating that Sx YCC3 and Lp MSZ2 could promote alcohol formation. Our result was in accordance with the findings that were reported by Chen et al. [10] who also found that lactic acid bacteria and *Staphylococcus* could promote the formation of alcohols. The possible reason for this result might lie in that the amino acids are converted into alcohols by the LAB in the sausage [42].

Esters are the source of fruity and caramel flavors in fermented sausages and they are derived from the reaction between acids and alcohols [14]. Esters are formed in the following two ways. The first is non-enzymatic catalytic esterification between alcohols and organic acids and the second is enzymatic catalytic esterification under the action of bacteria [48]. A total of 15 esters were detected within all of the fermented sausages. The concentration and categories of esters were both greater in the inoculated groups than in the CK group. Furthermore, carbonic acid ethyl hexadecyl ester, phenoxyethyl isobutyrate, formic acid hexyl ester, and ethyl caprate were detected only in the inoculated groups. Consistently, Stahnke et al. [49] reported that several esters were not contained in their control group, but they were detected in the fermented sausage that they had produced which was inoculated with *Staphylococcus xylosus*, indicating that *Staphylococcus xylosus* contained high-activity esterase and thus it could promote the production of esters.

A total of 14 acids were detected from within all of the fermented sausages. Acids are formed from carbohydrate degradation by bacteria. Acids have a strong odor and they are the precursors of other flavor compounds, such as ketones, alcohols, esters [14,42]. In this study, the concentration and categories of acids were significantly lesser in the CK group than in the inoculated groups, indicating that Lp MSZ2 and Sx YCC3 can promote the production of acids.

In summary, the concentrations of volatile compounds were higher in the inoculated groups than in the CK group. In addition, new volatile compounds (such as carbonic acid ethyl hexadecyl ester and phenoxyethyl isobutyrate) were detected in the inoculated sausages and they provided these sausages with a unique fermented flavor. Based on these findings, it could be concluded that inoculation with Sx YCC3 and Lp MSZ2 could promote volatile compound formation and improve the flavor of fermented sausage.

#### 3.6.2. Difference in the Volatile Composition

**Figure 5:**
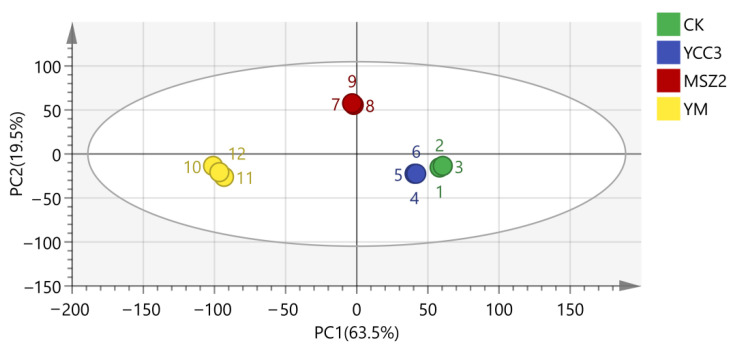
PCA of volatile flavor compounds in four fermented sausages at the end of ripening.

PCA, as an unsupervised clustering method, can provide an overview of the variations in volatile compounds that are present in fermented sausage. As shown in Figure 5, PC1 and PC2 explained 63.5% and 19.5% of the total variance, respectively. The volatile compounds of the four fermented sausages were located in different quadrants indicating that the four fermented sausages had a significant difference in their volatile compound compositions. This result might be attributed to the different categories of the inoculated bacteria in the fermented sausages. Chen et al. [10] reported that *Pediococcus pentosaceus*, *Lactobacillus curvatus*, *Lactobacillus sakei*, and *Staphylococcus xylosus*, when used as starter cultures, all improved the flavor of Harbin sausage and resulted in significant differences in the flavors among the groups.

#### 3.6.3. Identification of Differential Flavor Compounds from Fermented Sausages

**Figure 6:**
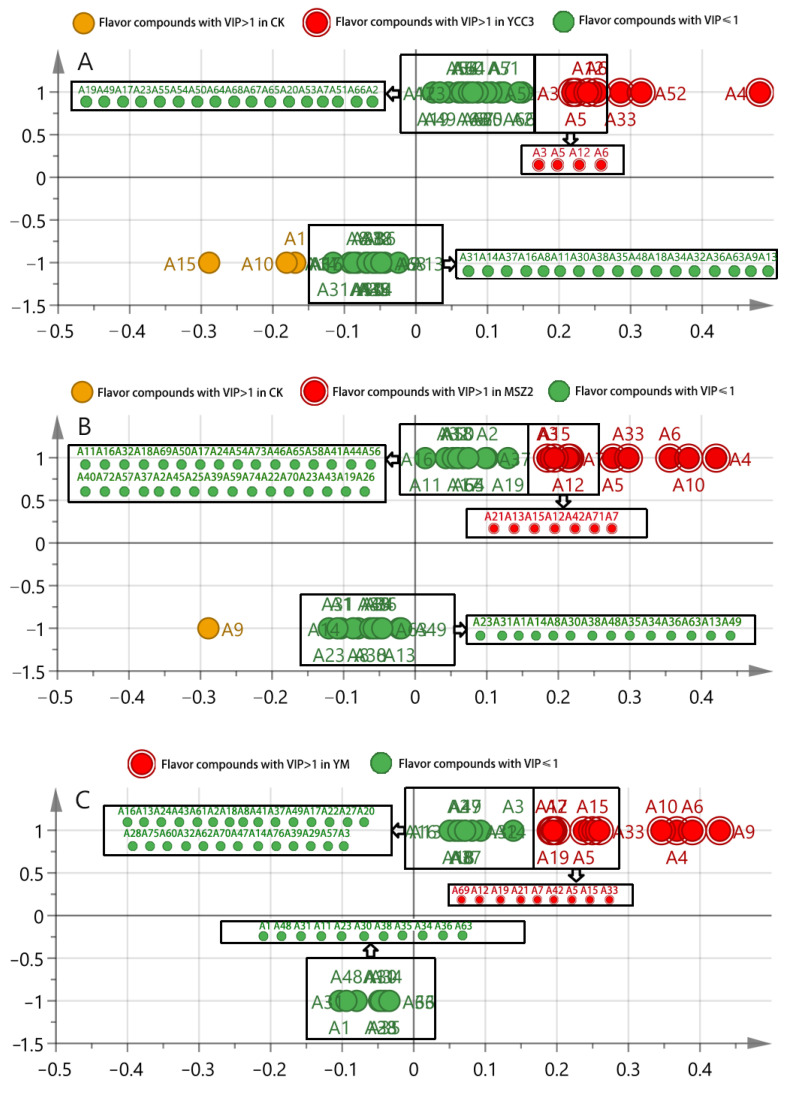
S-shaped loading plot obtained from OPLS-DA. (**A**) comparison between the CK and YCC3 groups; (**B**) comparison between the CK and MSZ2 groups; (**C**) comparison between the CK and YM groups.

OPLS-DA, as a multivariate calibration method, can be used in order to determine correlation through PCA so as to reduce the dimensionality of the datasets and it is also typically used for pairwise comparative analysis [50]. The flavor compounds with a VIP (variable importance in the projection) score that was >1 were defined as the significantly differential compounds. Heptaldehyde (A3), (E)-2-octenal (A5), octanal (A12), 1-nonanal (A6), 1-octen-3-ol (A33), benzoic acid, 2-[(trimethylsilyl)oxy], trimethylsilyl ester (A52), and (Z)-hept-2-enal (A4) were identified as the differential flavor compounds that were present in the YCC3 group but not in the CK group (Figure 6A). The differential flavor compounds that were present in the MSZ2 group were pentanal (A21), heptaldehyde (A3), (E,E)-2,4-decadienal (A15), octanal (A12), 2-nonenal (A7), (E)-2-octenal (A5), 2,4-dimethylpent-1-en-3-ol (A42), 1-octen-3-ol (A33), 1-nonanal (A6), 2-undecenal (A10), malonamic acid (A71), and (Z)-hept-2-enal (A4) (Figure 6B); these were not present in the CK group. The differential flavor compounds that were identified in the YM group included nonanoic acid (A69), octanal (A12), pentanal (A21), tetradecanal (A19), 2-nonenal (A7), 2,4-dimethylpent-1-en-3-ol (A42), (E)-2-octenal (A5), (E,E)-2,4-decadienal (A15), 1-octen-3-ol (A33), 2-undecenal (A10), (Z)-hept-2-enal (A4), 1-nonanal (A6), and 2-decenal (A9); these were not present in the CK group (Figure 6C). These results indicate that inoculation with different strains of bacteria, such as Sx YCC3 and Lp MSZ2, could produce various differential flavor compounds in sausages. Interestingly, the combined inoculation with Sx YCC3 and Lp MSZ2 resulted in the formation of new differential flavor compounds, rather than the superposition of the differential flavor compounds that were produced from the individual inoculations with Sx YCC3 alone or Lp MSZ2 alone. This might be due to the interaction between LAB and *Staphylococcus* which is able to produce new flavor compounds. The threshold values of 1-octen-3-ol (A33), octanal (A12), (E)-2-octenal (A5), 1-nonanal (A6), (E,E)-2,4-decadienal (A15), (Z)-hept-2-enal (A4), pentanal (A21), heptaldehyde (A3), and tetradecanal (A19) were 1, 0.7, 1, 3, 0.07, 13.5, 240, 3, and 53 (µg/kg in water), respectively. These flavor compounds play an important role in improving flavors. Compounds such as 1-octen-3-ol (A33), octanal (A12), (E)-2-octenal (A5), 1-nonanal (A6), (E,E)-2,4-decadienal (A15), (Z)-hept-2-enal (A4), pentanal (A21), heptaldehyde (A3), and tetradecanal can impart fat, lemon, green grass, citrus, fruit, mushroom, flower, tangerine, and other odors into sausage meat, thus they all have the potential to improve the flavor of fermented sausage [51]. These results indicate that inoculation with Sx YCC3 or Lp MSZ2 can improve sausage flavor, especially when the two inoculations are combined.

### 3.7. Correlation Analysis of Bacterial Relative Abundance, FFA Content, and Differential Flavor Compounds

**Figure 7:**
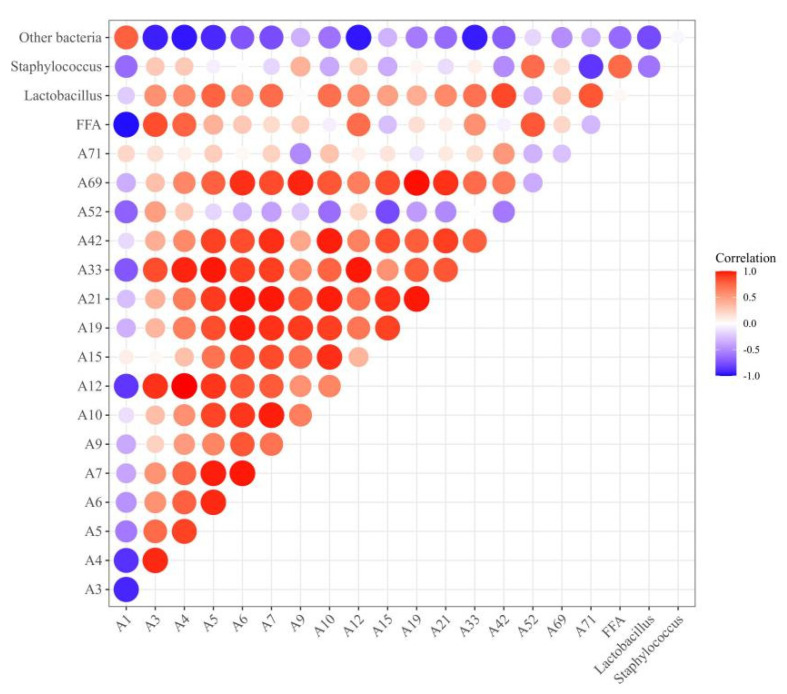
Heat map of correlation among bacterial relative abundance, FFA content, and differential flavor compounds. Positive correlation (0 < r < 1) and negative correlation (−1 < r < 0) were shown by red and blue color, respectively. A1: hexanal; A3: heptaldehyde; A4: (Z)-hept-2-enal; A5: (E)-2-octenal; A6: 1-nonanal; A7: 2-nonenal; A9: 2-decenal; A10: 2-undecenal; A12: octanal; A15: (E,E)-2,4-decadienal; A19: tetradecanal; A21: pentanal; A33: 1-octen-3-ol; A42: 2,4-dimethylpent-1-en-3-ol; A71: malonamic acid; A52: benzoic acid, 2-[(trimethylsilyl)oxy]-, trimethylsilyl ester; and A69: nonanoic acid.

The correlation analysis of bacterial relative abundance, the FFA content, and the differential flavor compounds is shown in Figure 7. The FFA content was positively correlated with the presence of *Staphylococcus* (r = 0.74). *Staphylococcus* has been reported to exhibit high lipase activity and it can promote the release of FFAs [43]. In this study, positive correlations were observed between the presence of FFAs and differential flavor compounds such as heptaldehyde (A3, r = 0.85), (Z)-hept-2-enal (A4, r = 0.77), octanal (A12, r = 0.73), and benzoic acid, 2-[(trimethylsilyl)oxy]-, trimethylsilyl ester (A52, r = 0.81), indicating that FFAs could promote the formation of flavor compounds since FFAs are the precursors of flavor compounds [10]. The relative abundance of *Lactobacillus* and *Staphylococcus* exhibited a positive correlation with differential flavor compounds. For example, the correlation coefficient between *Lactobacillus* and 2-nonenal (A7), 2-undecenal (A10), 1-octen-3-ol (A33), 2,4-dimethylpent-1-en-3-ol (A42), and malonamic acid (A71) were 0.73, 0.72, 0.71, 0.87, 0.82, respectively. The correlation coefficient between *Staphylococcus* and benzoic acid, 2-[(trimethylsilyl)oxy]-, trimethylsilyl ester (A52) and (Z)-hept-2-enal (A4) were 0.73 and 0.4, respectively. Our results were consistent with the findings that were reported by Ravyts et al. [52] and Hu et al. [42], who found a positive correlation between *S. xylosus* or LAB and volatile compounds within fermented sausage. In this study, *Lactobacillus* and *Staphylococcus* showed negative correlations with hexanal, whereas other bacteria exhibited positive correlations with hexanal (A1, r = 0.78).

Our correlation analysis indicated that the inoculation with Sx YCC3 or Lp MSZ2 changed the bacterial community composition and that after inoculation Sx YCC3 and Lp MSZ2 became the dominant bacteria. These two bacteria exhibited good lipid hydrolysis and antioxidation abilities, thus improving the flavor of the fermented sausages.

## 4. Conclusions

In this study, the analyses of bacterial community compositions showed that the inoculation of Lp MSZ2 and Sx YCC3 increased the competitiveness of *Lactobacillus* and *Staphylococcus* and inhibited the growth of unwanted bacteria. The analysis of the FFA content showed that Lp MSZ2 and Sx YCC3 promoted lipid hydrolysis. The investigation of the TBARS value, POV, and LOX activity indicated that inoculation with Lp MSZ2 or Sx YCC3 inhibited lipid oxidation. The amount of volatile flavor compounds was increased in the inoculated groups. The PCA and OPLS-DA showed that 1-octen-3-ol, octanal, (E)-2-octenal, 1-nonanal, and (Z)-hept-2-ena were common differential flavor compounds that were found in the inoculated sausages. Therefore, combined with the finding of our previous research, we suggest that MSZ2 and Sx YCC3 could be used as starter cultures for improving the quality of the bacteria, safety and flavor of fermented sausage.

## Figures and Tables

**Table 1:** Lipid composition in fermented sausage (g/100 g).

	Raw Meat	End of Ripening			
		CK	YCC3	MSZ2	YM
Neutral lipids	77.13 ± 0.78 ^a^	75.30 ± 0.99 ^a^	74.59 ± 0.60 ^a^	75.29 ± 0.75 ^a^	74.92 ± 0.10 ^a^
Phospholipids	18.16 ± 0.21 ^a^	12.15 ± 0.21 ^b^	7.01 ± 0.85 ^e^	10.58 ± 0.95 ^c^	8.92 ± 0.22 ^d^
Free fatty acids	4.71 ± 0.83 ^e^	12.55 ± 0.99 ^d^	18.40 ± 0.59 ^a^	14.13 ± 0.68 ^c^	16.16 ± 0.32 ^b^

Different letters in the same row indicate significant differences (*p* < 0.05). CK, MSZ2, YCC3, and YM represent the group without starter inoculation, with Lp MSZ2 inoculation, with Sx YCC3 inoculation, and with both bacterial inoculations, respectively.

**Table 2:** pH, POV, TBARS value, and LOX activity in fermented sausage during ripening process.

			Days of Ripening (d)		
	Batch	Raw Meat	0	6	12
pH	CK	5.78 ± 0.02 ^ax^	5.41 ± 0.02^cx^	5.41 ± 0.01 ^cx^	5.51 ± 0.01 ^bx^
	YCC3	5.78 ± 0.02 ^ax^	5.30 ± 0.02 ^cy^	5.23 ± 0.01 ^dy^	5.36 ± 0.01 ^by^
	MSZ2	5.78 ± 0.02 ^ax^	4.80 ± 0.01 ^bt^	4.70 ± 0.01 ^ct^	4.78 ± 0.01 ^bt^
	YM	5.78 ± 0.02 ^ax^	4.96 ± 0.02 ^cz^	4.90 ± 0.01 ^cz^	4.95 ± 0.01 ^bz^
POV (mmol/kg)	CK	0.83 ± 0.06 ^dx^	1.23 ± 0.01 ^cx^	2.87 ± 0.05 ^bx^	3.67 ± 0.04 ^ax^
	YCC3	0.83 ± 0.06 ^dx^	0.98 ± 0.01 ^cz^	1.99 ± 0.01 ^bt^	2.40 ± 0.03 ^at^
	MSZ2	0.83 ± 0.06 ^dx^	1.08 ± 0.01 ^cy^	2.61 ± 0.06 ^by^	2.83 ± 0.03 ^ay^
	YM	0.83 ± 0.06 ^dx^	0.91 ± 0.01 ^ct^	2.23 ± 0.04 ^bz^	2.66 ± 0.07 ^az^
TBARS value (mg/kg)	CK	0.11 ± 0.00 ^dx^	0.12 ± 0.00 ^cx^	0.31 ± 0.07 ^bx^	0.35 ± 0.02 ^ax^
	YCC3	0.11 ± 0.00 ^cx^	0.10 ± 0.00 ^cy^	0.13 ± 0.00 ^bt^	0.23 ± 0.02 ^az^
	MSZ2	0.11 ± 0.00 ^bx^	0.11 ± 0.01 ^by^	0.28 ± 0.07 ^ay^	0.27 ± 0.06 ^ay^
	YM	0.11 ± 0.00 ^cx^	0.10 ± 0.00 ^cy^	0.20 ± 0.01 ^bz^	0.25 ± 0.04 ^az^
LOX activity (U/g sample)	CK	74.00 ± 4.46 ^ax^	42.95 ± 1.44 ^bx^	27.51 ± 2.00 ^cx^	18.42 ± 0.36 ^dx^
	YCC3	74.00 ± 4.46 ^ax^	34.96 ± 0.21 ^by^	26.67 ± 0.71 ^cx^	15.99 ± 0.07 ^dy^
	MSZ2	74.00 ± 4.46 ^ax^	25.95 ± 0.37 ^bz^	17.23 ± 0.07 ^cy^	8.26 ± 0.11 ^dt^
	YM	74.00 ± 4.46 ^ax^	26.87 ± 0.91 ^bz^	18.26 ± 0.38 ^cy^	9.38 ± 0.25 ^dz^

a, b, c, d: different letters in the same row indicate significant differences (*p* < 0.05). x, y, z, t: different letters in the same column for each index indicate significant differences (*p* < 0.05)

**Table 3:** Counts of LAB, *Staphylococcus*, and *Enterobacteriaceae* (log CFU/g) in fermented sausage during ripening process.

			Days of Ripening (d)		
Bacterial	Batch	Raw Meat	0	6	12
LAB	CK	4.61 ± 0.02 ^dx^	7.08 ± 0.08 ^ct^	7.80 ± 0.04 ^az^	7.53 ± 0.05 ^by^
	YCC3	4.61 ± 0.02 ^dx^	7.42 ± 0.05 ^bz^	7.57 ± 0.05 ^ay^	7.17 ± 0.03 ^cz^
	MSZ2	4.61 ± 0.02 ^cx^	8.94 ± 0.04 ^sx^	8.88 ± 0.04 ^ax^	8.60 ± 0.05 ^bx^
	YM	4.61 ± 0.02 ^cx^	8.59 ± 0.04 ^by^	8.73 ± 0.05 ^ay^	8.69 ± 0.04 ^ax^
*Staphylococcus*	CK	4.21 ± 0.02 ^dx^	7.42 ± 0.05 ^az^	6.89 ± 0.03 ^bz^	6.72 ± 0.05 ^cz^
	YCC3	4.21 ± 0.02 ^dx^	8.04 ± 0.05 ^ay^	8.96 ± 0.01 ^bx^	8.82 ± 0.02 ^cx^
	MSZ2	4.21 ± 0.02 ^cx^	6.79 ± 0.03 ^at^	6.76 ± 0.03 ^at^	6.55 ± 0.09 ^bt^
	YM	4.21 ± 0.02 ^dx^	7.91 ± 0.02 ^ay^	7.84 ± 0.03 ^by^	7.64 ± 0.04 ^cy^
*Enterobacteriaceae*	CK	4.04 ± 0.05 ^dx^	5.67 ± 0.03 ^bx^	5.98 ± 0.01 ^ax^	5.52 ± 0.06 ^cx^
	YCC3	4.04 ± 0.05 ^cx^	5.24 ± 0.04 ^ay^	4.63 ± 0.07 ^byz^	4.11 ± 0.09 ^cz^
	MSZ2	4.04 ± 0.05 ^cx^	5.18 ± 0.02 ^ay^	4.71 ± 0.05 ^by^	4.58 ± 0.07 ^by^
	YM	4.04 ± 0.05 ^dx^	4.73 ± 0.09 ^az^	4.55 ± 0.07 ^bz^	4.24 ± 0.01 ^cz^

a, b, c, d: different letters in the same row indicate significant differences (*p* < 0.05). x, y, z, t: different letters in the same column for each index indicate significant differences (*p* < 0.05).

**Table 4:** Content of volatile flavor compound (ug/kg) in four fermented sausage samples.

Volatile Compound	RT	CK	YCC3	MSZ2	YM
Hexanal (A1)	5.72	304.81 ± 11.40 ^a^	181.89 ± 3.91 ^d^	253.75 ± 7.07 ^b^	207.76 ± 6.17 ^c^
trans-2-Hexenal (A2)	7.42	38.92 ± 5.68 ^c^	134.28 ± 2.87 ^a^	86.90 ± 3.66 ^b^	74.33 ± 5.04 ^b^
Heptaldehyde (A3)	9.13	74.04 ± 1.93 ^c^	279.59 ± 10.38 ^a^	234.82 ± 8.18 ^a^	259.69 ± 17.02 ^ab^
(Z)-Hept-2-enal (A4)	11.22	212.80 ± 9.74 ^d^	1210.64 ± 16.77 ^b^	1052.60 ± 24.56 ^c^	1463.59 ± 29.35 ^a^
(E)-2-Octenal (A5)	14.69	111.68 ± 3.22 ^d^	326.70 ± 8.81 ^c^	474.92 ± 15.69 ^b^	637.82 ± 20.71 ^a^
1-Nonanal (A6)	16.92	916.26 ± 14.34 ^d^	1192.00 ± 26.48 ^c^	1518.29 ± 33.28 ^b^	2311.95 ± 40.11 ^a^
2-Nonenal (A7)	19.00	150.87 ± 6.97 ^d^	211.47 ± 8.78 ^c^	372.84 ± 9.33 ^b^	516.98 ± 10.36 ^a^
(Z)-4-Decenal (A8)	20.37	29.46 ± 0.88 ^b^	n.d.	n.d.	71.90 ± 1.01 ^a^
2-Decenal (A9)	22.66	557.00 ± 34.12 ^b^	548.82 ± 20.64 ^b^	159.92 ± 7.68 ^c^	2249.98 ± 40.66 ^a^
2-Undecenal (A10)	26.14	794.71 ± 17.68 ^c^	653.05 ± 10.12 ^d^	1488.17 ± 53.11 ^b^	1900.83 ± 48.42 ^a^
Benzylcarboxaldehyde (A11)	14.59	21.14 ± 0.12 ^a^	n.d.	21.99 ± 0.08 ^a^	n.d.
Octanal (A12)	13.02	168.38 ± 10.84 ^c^	417.43 ± 15.66 ^b^	387.40 ± 14.51 ^b^	506.59 ± 17.33 ^a^
Decanal (A13)	20.69	45.54 ± 2.52 ^b^	42.91 ± 2.13 ^b^	43.17 ± 1.84 ^b^	75.10 ± 3.28 ^a^
2,4-Nonadienal (A14)	21.05	36.25 ± 1.25 ^b^	n.d.	n.d.	115.13 ± 2.36 ^a^
(E,E)-2,4-decadienal (A15)	23.79	358.31 ± 5.68 ^c^	n.d.	537.94 ± 10.33 ^b^	935.40 ± 12.56 ^a^
Undecanal (A16)	24.24	30.51 ± 0.64 ^c^	n.d.	39.13 ± 1.72 ^b^	52.66 ± 2.34 ^a^
Dodecyl aldehyde (A17)	27.59	80.59 ± 1.38 ^d^	89.12 ± 2.75 ^c^	102.51 ± 3.52 ^b^	128.60 ± 1.88 ^a^
Lily aldehyde (A18)	31.22	13.59 ± 0.22 ^c^	n.d.	29.34 ± 1.39 ^b^	49.39 ± 3.15 ^a^
Tetradecanal (A19)	39.39	25.10 ± 0.17 ^c^	27.59 ± 1.01 ^b^	106.09 ± 3.66 ^b^	375.24 ± 5.84 ^a^
2,4-Decadienal (A20)	23.90	n.d.	42.22 ± 2.41 ^b^	n.d.	54.88 ± 1.28 ^a^
Pentanal (A21)	3.41	n.d.	n.d.	157.44 ± 5.38 ^b^	357.70 ± 6.32 ^a^
cis-9-Tetradecenal (A22)	30.88	n.d.	n.d.	57.32 ± 2.27 ^a^	49.54 ± 1.64 ^b^
Nerylacetone (A23)	28.78	69.42 ± 3.62 ^b^	84.42 ± 2.31 ^a^	n.d.	49.54 ± 2.36 ^c^
2-Heptanone (A24)	8.69	n.d.	n.d.	26.26 ± 2.22 ^b^	31.91 ± 1.34 ^a^
5-Decanone (A25)	22.06	n.d.	n.d.	53.20 ± 1.61 ^a^	n.d.
Geranylacetone (A26)	28.76	n.d.	n.d.	88.90 ± 4.39 ^a^	n.d.
1-Octen-3-one (A27)	12.00	n.d.	n.d.	n.d.	50.14 ± 1.16 ^a^
2-Undecanone (A28)	23.70	n.d.	n.d.	n.d.	55.76 ± 1.27 ^a^
2-Tridecanone (A29)	30.25	n.d.	n.d.	n.d.	117.02 ± 2.10 ^a^
2,6-Octadien-1-ol (A30)	26.53	19.39 ± 1.00 ^a^	n.d.	n.d.	n.d.
2,3-Butanediol (A31)	5.52	57.04 ± 2.02 ^a^	n.d.	n.d.	n.d.
1-Hexanol (A32)	8.07	13.28 ± 0.39 ^c^	n.d.	26.45 ± 2.34 ^b^	75.09 ± 1.79 ^a^
1-Octen-3-ol (A33)	12.17	94.43 ± 1.88 ^d^	449.92 ± 11.97 ^c^	518.80 ± 15.12 ^b^	719.48 ± 20.39 ^a^
(Z)-5-Octen-1-ol (A34)	13.39	13.57 ± 0.61 ^a^	n.d.	n.d.	n.d.
1-Octyn-3-ol (A35)	14.69	15.85 ± 0.54 ^a^	n.d.	n.d.	n.d.
(2Z)-2-Octene-1-ol (A36)	17.30	10.74 ± 0.17 ^a^	n.d.	n.d.	n.d.
2-Pentadecyn-1-ol (A37)	25.62	31.87 ± 0.35 ^b^	n.d.	76.79 ± 1.49 ^a^	78.11 ± 0.92 ^a^
2,6-Octadien-1-ol (A38)	26.53	19.39 ± 0.08 ^a^	n.d.	n.d.	n.d.
1-Pentanol (A39)	4.91	n.d.	n.d.	56.68 ± 2.37 ^b^	110.41 ± 3.16 ^a^
2-Methyl-1-butanol (A40)	5.15	n.d.	n.d.	32.62 ± 1.69 ^a^	n.d.
n-Heptanol (A41)	11.84	n.d.	n.d.	28.96 ± 0.54 ^b^	43.72 ± 1.58 ^a^
2,4-Dimethylpent-1-en-3-ol (A42)	16.55	n.d.	n.d.	374.42 ± 9.34 ^b^	407.25 ± 10.47 ^a^
Phenylethyl Alcohol (A43)	17.39	n.d.	n.d.	71.63 ± 2.94 ^a^	32.78 ± 2.96 ^b^
2,4-Decadien-1-ol (A44)	20.36	n.d.	n.d.	29.14 ± 1.94 ^a^	n.d.
trans-2-Octen-1-ol (A45)	26.46	n.d.	n.d.	52.76 ± 1.83 ^a^	n.d.
2-Hexadecanol (A46)	23.38	n.d.	n.d.	26.74 ± 0.28 ^a^	n.d.
2-Octyldecanol (A47)	32.11	n.d.	n.d.	n.d.	77.23 ± 1.06 ^a^
4-tert-Butylcyclohexyl acetate (A48)	24.99	81.36 ± 2.05 ^a^	66.98 ± 3.04 ^b^	65.19 ± 1.75 ^b^	n.d.
Propanoic acid, 2-methyl-, 1-(1,1-dimethylethyl) -2-methyl-1,3-propanediyl ester (A49)	33.18	26.25 ± 1.41 ^c^	31.33 ± 1.06 ^c^	24.55 ± 1.32 ^c^	72.95 ± 1.85 ^a^
3,6-Octadecadiynoic acid, methyl ester (A50)	13.40	n.d.	22.15 ± 0.64 ^a^	17.19 ± 0.85 ^b^	n.d.
Octaethylene glycol monododecyl ether (A51)	6.20	n.d.	64.77 ± 2.53 ^a^	n.d.	n.d.
Benzoic acid, 2-[(trimethylsilyl)oxy]-, trimethylsilyl ester (A52)	18.13	n.d.	428.85 ± 12.35 ^a^	n.d.	n.d.
4-Hexenoic acid, 6-hydroxy-4-methyl-, methyl ester, (E)-(A53)	13.40	n.d.	47.72 ± 1.24 ^a^	n.d.	n.d.
Cyclopropanetetradecanoic acid, 2-octyl-, methyl ester (A54)	28.32	n.d.	18.61 ± 0.52 ^b^	26.36 ± 1.07 ^a^	n.d.
Formic acid, hexyl ester (A55)	8.07	n.d.	18.12 ± 0.96 ^a^	n.d.	n.d.
2′-Hexyl-1,1′-bicyclopropane-2-octanoic acid methyl ester (A56)	22.17	n.d.	n.d.	30.71 ± 1.04 ^a^	n.d.
4-Hydroxynonanoic acid gamma-lactone (A57)	25.95	n.d.	n.d.	43.24 ± 2.11 ^b^	129.20 ± 1.97 ^a^
Phenoxyethyl isobutyrate (A58)	30.89	n.d.	n.d.	28.19 ± 0.38 ^a^	n.d.
Carbonic acid, ethyl hexadecyl ester (A59)	31.41	n.d.	n.d.	56.82 ± 1.29 ^a^	n.d.
(E)-9-Tetradecen-1-olacetate (A60)	24.99	n.d.	n.d.	n.d.	56.13 ± 1.05 ^a^
Ethyl caprate (A61)	27.11	n.d.	n.d.	n.d.	35.18 ± 1.11 ^a^
Crotonic acid, methyl ester (A62)	29.66	n.d.	n.d.	n.d.	63.32 ± 2.76 ^a^
3-Hydroxydodecanoic acid (A63)	23.54	10.33 ± 0.56 ^a^	n.d.	n.d.	n.d.
Allantoic acid (A64)	1.51	n.d.	23.07 ± 0.31 ^a^	n.d.	n.d.
Undec-10-ynoic acid (A65)	20.28	n.d.	42.14 ± 0.64 ^a^	26.95 ± 1.31 ^b^	n.d.
trans-2-Hexenoic acid (A66)	26.72	n.d.	89.67 ± 1.36 ^a^	n.d.	n.d.
2-Hexenoic acid (A67)	26.88	n.d.	31.89 ± 1.02 ^a^	n.d.	n.d.
trans-2-undecenoic acid (A68)	27.12	n.d.	27.23 ± 0.09 ^a^	n.d.	n.d.
Nonanoic acid (A69)	23.29	n.d.	n.d.	16.99 ± 0.39 ^b^	242.59 ± 9.46 ^a^
7-Nonynoic acid (A70)	12.82	n.d.	n.d.	62.36 ± 2.66 ^b^	72.87 ± 1.94 ^a^
Malonamic acid (A71)	1.96	n.d.	n.d.	825.88 ± 13.58 ^a^	n.d.
3-Decenoic acid (A72)	23.37	n.d.	n.d.	36.37 ± 0.22 ^a^	n.d.
7-Oxooctanoic acid (A73)	30.26	n.d.	n.d.	26.74 ± 1.23 ^a^	n.d.
Erucic acid (A74)	33.41	n.d.	n.d.	57.32 ± 2.36 ^a^	n.d.
Octanoic acid (A75)	19.81	n.d.	n.d.	n.d.	55.81 ± 0.99 ^a^
n-Decanoic acid (A76)	23.36	n.d.	n.d.	n.d.	109.79 ± 2.37 ^a^

n.d.: volatile flavor compounds not detected. a–d: different letters in the same row indicate significant differences (*p* < 0.05). A1–A76: following the name of volatile compound represents the number of flavor substances. RT: retention time (min).

## Data Availability

The data that are presented in this study are available on request from the corresponding author.
